# Impact of ursodeoxycholic acid treatment on Fontan-associated liver disease

**DOI:** 10.1007/s00535-024-02168-x

**Published:** 2024-11-27

**Authors:** Tomomi Kogiso, Yuri Ogasawara, Makiko Taniai, Eriko Shimada, Kei Inai, Katsutoshi Tokushige, Yousuke Nakai

**Affiliations:** 1 Institute of Gastroenterology, Department of Internal Medicine, Tokyo Women’s Medical University, 8-1 Kawada-cho, Shinjuku-ku, Tokyo, 162-8666 Japan; 2 Pediatric cardiology and adult congenital cardiology, Tokyo Women’s Medical University, 8-1 Kawada-cho, Shinjuku-ku, Tokyo, 162-8666 Japan; 3 Japan Community Health care Organization Tokyo Joto Hospital, 9-13-1 Kameido, Koto-ku, Tokyo, 136-0071 Japan

**Keywords:** Fontan procedure, Fontan-associated liver disease, Hepatocellular carcinoma, Ursodeoxycholic acid

## Abstract

**Background:**

Fontan-associated liver disease (FALD) is a type of progressive liver fibrosis that occurs following Fontan surgery and can be complicated by hepatocellular carcinoma (HCC). Established treatments for FALD are lacking. Therefore, we investigated the efficacy of ursodeoxycholic acid (UDCA) in patients with FALD.

**Methods:**

This single-center retrospective study was conducted from 2003 to 2024 and involved 220 patients (103 men, 46.8%) who had been diagnosed with FALD. UDCA was administered to 113 patients presenting with liver or biliary enzyme abnormalities. We evaluated the patients’ liver enzyme levels 3, 6, and 12 months after treatment. HCC developed in 10.5% and the mortality rate was 4.5%. Survival and cumulative incidence of HCC were compared between patients with and without UDCA treatment using Kaplan–Meier curves and propensity-matched analysis (*n* = 68 per group).

**Results:**

UDCA treatment significantly reduced the aspartate aminotransferase (AST), alanine transaminase (ALT), and gamma-glutamyl transferase (GGT) levels at 3 months. The mean pretreatment AST/ALT/GGT levels were 26/22/323 U/L, respectively, and decreased to 19/15/102 U/L at 3 months, 18/12/88 U/L at 6 months, and 16/19/64 U/L at 12 months. However, the total bilirubin level and platelet count did not show significant differences. The survival rate was higher and the HCC rate was lower in patients with than without UDCA treatment. The 5-year incidence rate of HCC was 5.6% in the UDCA group and 24.2% in the untreated group.

**Conclusions:**

UDCA treatment significantly reduced liver enzyme levels, including GGT, and mitigated the progression of HCC. UDCA may be beneficial for patients with FALD.

## Introduction

Fontan-associated liver disease (FALD) is characterized by progressive liver fibrosis following Fontan surgery and is associated with complications such as focal nodular hyperplasia and hepatocellular carcinoma (HCC) [1, 2]. The incidence rates of FALD vary widely, ranging from 36% to 86% [3, 4], and liver damage tends to worsen over time after the Fontan procedure. Although there are no universally accepted diagnostic criteria for FALD, the European Association for the Study of the Liver and the European Reference Networks (EASL-ERN) has outlined its key characteristics [5]. The gamma-glutamyl transferase (GGT) and total bilirubin (T-BIL) levels are frequently elevated in patients with FALD. Our national survey, which involved 1,666 patients from multiple centers, showed that the GGT level was elevated in 40% of patients [6].

GGT is an early predictive marker for atherosclerosis, heart failure, arterial stiffness, and plaque formation [7, 8]. It is also an important surrogate marker for the development of FALD [9]. We recently reported that an elevated GGT level is associated with a more rapid decline in the platelet count, suggesting higher risk for liver fibrosis [10].

Regarding liver tumors, a nationwide study in Japan reported HCC in 31 of 2,700 (1.15%) patients [11]. We previously reported that the incidence of HCC increased to 0.8% and 2.9% 10 and 20 years after the Fontan procedure, respectively [12]. High central venous pressure and severe atrioventricular valve regurgitation are associated with the development of HCC [13]. In addition, complications such as polysplenia, a higher model for end-stage liver disease excluding the International normalized ratio (MELD-XI) score [14], the absence of warfarin treatment, and situs inversus [15] are independent risk factors for HCC development. Managing FALD and preventing HCC remain urgent clinical challenges.

No treatment has been established for FALD. In a previous study, we found that GGT levels significantly improved in patients treated with ursodeoxycholic acid (UDCA) [10]. UDCA is a hepatoprotective agent with multiple beneficial effects, including the improvement of bile flow, dissolution of gallstones, enhancement of liver perfusion, protection of hepatocytes, and facilitation of fat digestion [16]. It is commonly used in chronic liver diseases, such as primary biliary cholangitis, and it reduces serum levels of liver and biliary enzyme levels [17, 18]. Although diarrhea is a possible side effect, severe adverse effects are rare, which makes UDCA a suitable option for improving liver function in patients with chronic liver diseases. However, the efficacy of UDCA in patients with FALD has not been well studied.

In this study, we evaluated the efficacy of UDCA treatment in patients with FALD by monitoring liver and biliary enzyme levels and comparing survival and HCC incidence between those who were and were not treated with UDCA.

## Methods

### Patients and study design

**Fig. 1:**
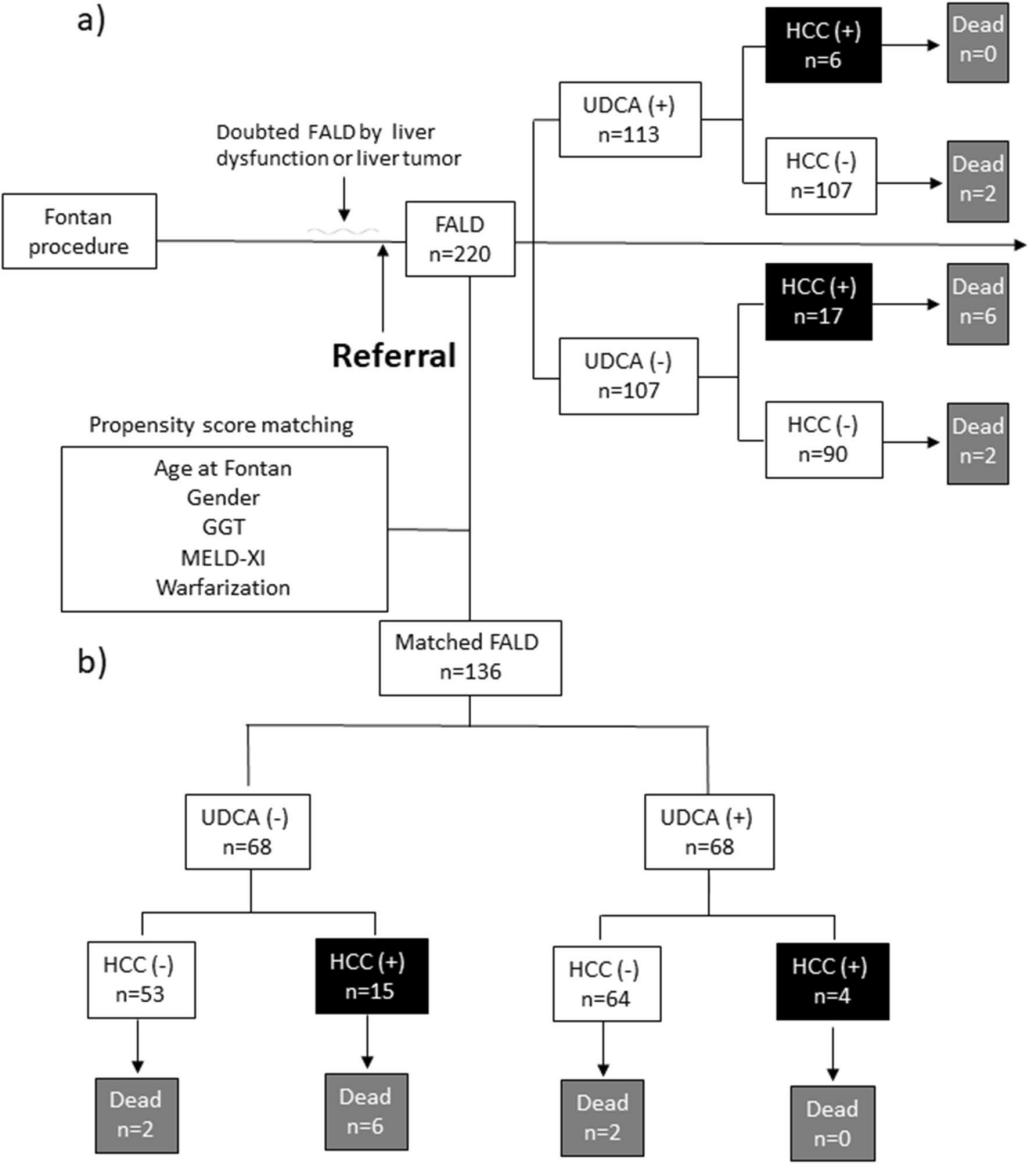

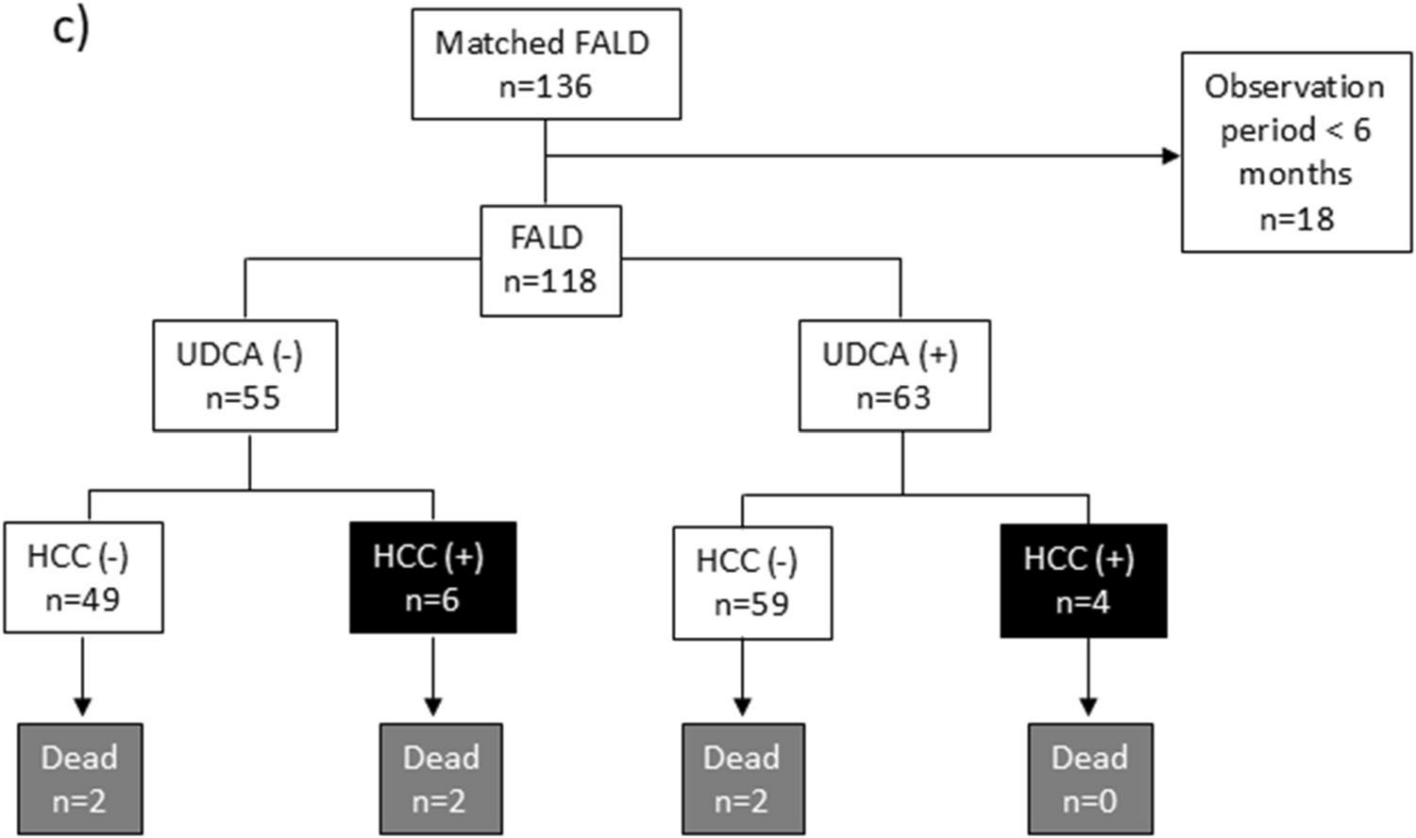
Flowchart of UDCA-treated and untreated patients with FALD and their outcomes. **a** Flowchart of the total cohort of 220 patients with FALD referred to our department. UDCA treatment was administered to 113 patients. HCC was observed in 17 untreated patients and in 6 UDCA-treated patients. **b** Flowchart of patients with FALD after propensity matching. HCC was observed in 15 untreated patients and in 4 UDCA-treated patients. **c** Flowchart of patients with FALD with ≥6 months of follow-up. HCC was observed in 6 untreated patients and in 4 UDCA-treated patients. *FALD* Fontan-associated liver disease, *GGT* gamma-glutamyl transferase, *HCC* hepatocellular carcinoma, *MELD-XI* model for end-stage liver disease excluding the international normalized ratio, *UDCA* ursodeoxycholic acid

This study was retrospectively conducted by single-center, an observational analysis. It involved 220 patients diagnosed with FALD who were referred to our department between 2003 and 2024 for liver damage or space-occupying lesions of the liver (Fig. 1). Demographic information and laboratory data were collected at the time of UDCA treatment initiation or during the first visit to our department. UDCA was administered at a dose of 150 to 600 mg/day to patients with liver or biliary enzyme abnormalities, including elevated GGT levels with agreement. In patients with diarrhea, the UDCA dose was started at less than 600 mg/day, and intestinal regulators were prescribed if needed. FALD was diagnosed based on liver structural abnormalities and elevated liver enzyme or T-BIL levels attributable to the Fontan circulation [19]. Patients were followed up every 3 to 6 months with blood tests and/or imaging studies.

We assessed the efficacy of UDCA treatment by monitoring the following parameters:

- Liver enzyme levels (T-BIL, aspartate aminotransferase [AST], alanine transaminase [ALT], and GGT) and platelet counts 3, 6, and 12 months after treatment
- Liver enzyme levels and platelet counts before and after treatment, as well as at the first and last visits, between UDCA-treated and untreated patients
- Survival rates between UDCA-treated and untreated patients after propensity matching for confounding variables, including age at Fontan surgery, sex, GGT level, MELD-XI score, and warfarin use
- HCC occurrence between UDCA-treated and untreated patients, considering both the overall study population and those treated ≥ 6 months

The study was conducted in accordance with the principles of the Declaration of Helsinki and the ethical guidelines of Tokyo Women’s Medical University Hospital. The Institutional Review Board of Tokyo Women’s Medical University Hospital approved the study protocol, and informed consent was obtained from all the participants on an opt-out basis given the retrospective nature of the study.

### Clinical parameters

The following clinical parameters were collected: age, sex, date of the Fontan procedure, congenital complications, primary cardiac disease, HCC incidence, survival rate, and use of warfarin potassium, and aspirin. Laboratory parameters measured at the first visit or at the time of UDCA treatment included serum levels of albumin, T-BIL, AST, ALT, GGT, the prothrombin, international normalized ratio (INR), platelet count, brain natriuretic peptide (BNP), alpha-fetoprotein, and the hepatitis B surface antigen and anti-hepatitis C virus antibody status. Patients with protein-losing enteropathy were excluded from the albumin level assessments. Those prescribed warfarin were excluded from the prothrombin and INR evaluations. Liver fibrosis markers, including hyaluronic acid (ng/mL) and type IV collagen 7S (ng/mL), were also measured [19].

### Diagnosis of HCC

HCC was diagnosed based on histological examination or imaging studies, including abdominal ultrasound, abdominal enhanced computed tomography, and/or gadolinium ethoxybenzyl diethylenetriamine penta-acetic acid-enhanced magnetic resonance imaging (Bayer Schering Pharma, Berlin, Germany), along with elevated alpha-fetoprotein levels [20, 21]. HCC staging was evaluated by the Union for International Cancer Control (UICC) stage (8th edition) [22].

### Statistical analysis

Data are presented as medians with ranges in normal distribution and means with standard deviations in non-normal distributions. Categorical variables were compared between UDCA-treated and untreated patients using the Mann–Whitney *U* test and the *χ*^2^ test, performed with SPSS software version 29.0.1 (IBM Corp., Armonk, NY, USA). Statistical significance was set at *p* < 0.05. A propensity score for the predicted probability of UDCA treatment in each patient was estimated with the use of a logistic-regression model fit with the 5 factors. We created a propensity score-matched cohort by attempting to match each patient who received UDCA treatment with one who non-treated (a 1:1 match). The logit of their propensity score, with matching occurring if the difference in the logits of the propensity scores was less than 0.25 times the standard deviation of the scores (the caliper width). The survival rate and HCC incidence were evaluated using Kaplan–Meier curves after propensity score matching, and the log-rank test was used to estimate differences according to the UDCA treatment status. Cox regression analysis was conducted to assess the impact of UDCA treatment on HCC development and survival, adjusting for potential confounding variables including age at Fontan surgery, sex, GGT level, MELD-XI score, (HCC for survival) and warfarin treatment. Hazard ratios (HRs) and 95% confidence intervals (CIs) were calculated.

## Results

### Characteristics of UDCA-treated and untreated patients with FALD

Of the 220 patients who underwent the Fontan procedure, 113 were treated with UDCA at a dose of 150 to 600 mg/day due to liver or biliary enzyme abnormalities (Table 1). A flowchart of UDCA-treated and untreated patients with FALD and their outcomes is shown in Figure 1. The median age at the time of Fontan surgery and UDCA treatment was 26.7 years (range, 12.3–57.2 years) and 5.6 years (range, 0.9–37.5 years), respectively, in UDCA-treated patients and 25.9 years (range, 9.3–47.1 years) and 4.6 years (range, 0.0–29.7 years) in untreated patients. UDCA-treated patients tended to be older at the time of treatment and Fontan surgery than untreated patients (both *p* = 0.07). Men were significantly more likely to receive UDCA treatment (*p* < 0.01). Underlying cardiac disease and complications such as asplenia, polysplenia, visceral inversion/confusion, PLE, and hepatitis viruses were not significantly different between the two groups. HCC was more common in patients who did not receive UDCA treatment (17 [15.9%] vs. 6 [5.3%], *p* = 0.01). In terms of the characteristics of FALD-HCC, maximum diameter was 36 (11-80) mm in UDCA (-), 19 (15-25) in UDCA treatment, number of nodules of 1, 2, and ≥ 3 was 10/3/4 and 5/0/1, respectively (Table 2). The stage of HCC (IA/IB/II/IIIA/IIIB/IVA/IVB) was 5/5/5/1/1/0/0 and 2/2/0/0/1/0/1, respectively (*p* = 0.19). Initial treatment for HCC included liver resection in 2 cases, transcatheter arterial chemo-embolization and proton beam therapy in 6 cases, and radiation in 5 cases, and chemotherapy in 1 case; however, 3 cases received best supportive care due to cardiac disease. There was no significant difference between UDCA-treated and non-treated patients regarding the HCC status. The use of antiplatelet agents was similar between the groups but anticoagulant use was significantly higher in UDCA-treated patients (*p* = 0.02). Mild diarrhea was the only side effect observed in patients receiving UDCA.

**Table 1:** Characteristics of patients with FALD

	Total (*n* = 220)	Pre-matching			Propensity score matching		
		UDCA (–) (*n* = 107)	UDCA (+) (*n* = 113)	*p*-value [UDCA (–) vs. (+)]	UDCA (–) (*n* = 68)	UDCA (+) (*n* = 68)	*p*-value [UDCA (–) vs. (+)]
Age at time of UDCA treatment or referral, years	26.1 (9.3–57.2)	25.9 (9.3–47.1)	26.7 (12.3–57.2)	0.07	26.5 (13.7–47.1)	27.7 (12.3–53.0)	0.35
Age at time of Fontan operation, years	5.1 (0.0–37.5)	4.6 (0.0–29.7)	5.6 (0.9–37.5)	0.07	5.2 (0.0–29.7)	6.4 (0.9–37.5)	0.63
Men	103 (46.8)	40 (37.4)	63 (55.8)	<0.01	34 (50.0)	29 (42.6)	0.39
Cardiac disease							
Single cardiac ventricle	80 (36.3)	40 (37.4)	40 (35.4)	0.62	25 (36.8)	28 (41.2)	0.48
Pulmonary atresia	20 (9.1)	8 (7.5)	12 (10.6)		4 (5.9)	9 (13.2)	
Tricuspid valve insufficiency	50 (22.7)	26 (24.3)	24 (21.2)		17 (25.0)	16 (23.5)	
Double-outlet right ventricle	63 (28.6)	32 (29.9)	31 (27.4)		24 (35.3)	15 (22.1)	
Transposition of the great arteries	23 (10.5)	9 (8.4)	14 (12.4)		4 (5.9)	5 (7.4)	
Hypoplastic left heart syndrome	31 (14.1)	13 (12.1)	18 (15.9)		7 (10.3)	10 (14.7)	
Others	9 (4.1)	5 (4.7)	4 (3.5)		3 (4.4)	3 (4.4)	
Complications							
Asplenia	21 (9.5)	7 (6.5)	14 (12.4)	0.14	7 (10.3)	6 (8.8)	0.77
Polysplenia	17 (7.7)	9 (8.4)	8 (7.1)	0.71	7 (10.3)	4 (5.9)	0.35
Visceral inversion/confusion	25/7 (14.5)	9/3 (11.2)	16/4 (17.7)	0.17	6/2 (11.8)	13/2 (22.1)	0.11
PLE	7 (3.2)	5 (4.7)	2 (1.8)	0.22	4 (5.9)	2 (2.9)	0.78
HBs antigen-positive	1 (0.5)	0 (0.0)	1 (0.9)	0.33	0 (0.0)	1 (1.5)	0.32
HCV antibody-positive	6 (2.7)	4 (3.7)	2 (1.8)	0.37	3 (4.4)	1 (1.5)	0.31
HCC	23 (10.5)	17 (15.9)	6 (5.3)	0.01	15 (22.1)	4 (5.9)	<0.01
Treatment							
Warfarin	146 (66.4)	63 (58.9)	83 (73.5)	0.02	51 (75.0)	47 (74.6)	0.44
Aspirin	135 (61.4)	69 (64.5)	66 (58.4)	0.35	40 (58.8)	40 (58.8)	1.00

Data are presented as median (range) or n (%)

*FALD* Fontan-associated liver disease, *HBs antigen* hepatitis B surface antigen, *HCC* hepatocellular carcinoma, *HCV* hepatitis C virus, *PLE* protein-losing enteropathy, UDCA ursodeoxycholic acid

**Table 2:** Characteristics of FALD-HCC

	Total (n=23)	Pre-matching			Propensity score matching (observation ≥ 6 months)		
		UDCA (–) (*n* = 17)	UDCA (+) (*n* = 6)	*p*-value [UDCA (–) vs. (+)]	UDCA (–) (*n* = 6)	UDCA (+) (*n* = 4)	*p*-value [UDCA (–) vs. (+)]
Age at Fontan procedure, years	7.2 (1.4-25.6)	6.9 (1.4-25.6)	8.2 (5.5-14.0)	0.51	6.7 (4.9-10.6)	8.7 (6.2-14.0)	0.25
Age at HCC, years	32.6 (19.7-45.4)	31.0 (19.7-45.4)	36.9 (26.8-43.3)	0.08	32.3 (22.3-36.5)	36.5 (26.8-42.5)	0.43
Men	12 (52.2)	9 (52.9)	3 (50.0)	0.46	3 (50.0)	2 (50.0)	1.00
Maximum diameter (mm)	22 (11-80)	36 (11-80)	19 (15-25)	0.03	29 (11-60)	20 (15-22)	0.33
Number of nodules (1/2/ ≥ 3)	15/3/5	10/3/4	5/0/1	0.46	3/2/1	3/0/1	0.43
Stage (IA/IB/II/IIIA/IIIB/IVA/IVB)	7/7/5/1/2/0/1	5/5/5/1/1/0/0	2/2/0/0/1/0/1	0.19	IA 2/IB 1/II 2/IIIA 1	IA 1 /IB 1 /IIIB 1/IVB 1	0.40
Treatment*							
Hepatic resection	2 (8.7)	2 (11.7)	0 (0.0)	0.65	1 (16.7)	0 (0.0)	0.83
TACE	6 (26.1)	4 (23.5)	2 (33.3)		1 (16.7)	1 (25.0)	
Proton beam therapy	6 (26.1)	5 (29.4)	1 (16.7)		1 (16.7)	1 (25.0)	
Stereotactic radio therapy	5 (21.7)	3 (17.6)	2 (33.3)		2 (33.3)	1 (20.0)	
Chemotherapy	1 (4.3)	0 (0.0)	1 (16.7)		0 (0.0)	1 (25.0)	
BSC	3 (13.0)	3 (17.6)	0 (0.0)		1 (16.7)	0 (0.0)	
Prognosis, death	6 (26.1)	6 (35.3)	0 (0.0)	0.09	2 (33.3)	0 (0.0)	0.20

Data are presented as median (range) or n (%)

*BSC* best supportive care, *FALD* Fontan-associated liver disease, *HCC* hepatocellular carcinoma, *TACE* transcatheter arterial chemo-embolization, *UDCA* ursodeoxycholic acid

^*^Initial treatment of HCC

In the initial blood tests conducted at the time of UDCA treatment or the first visit to our department (Table 3), GGT levels were significantly higher in UDCA-treated patients (114 vs. 60 U/L, *p* < 0.01). Type IV collagen 7S levels were slightly elevated in UDCA-treated patients (7.5 vs. 8.0 ng/mL, *p* = 0.07). There were no significant differences in other laboratory data, including albumin levels, BNP levels, and MELD-XI scores.

**Table 3:** Laboratory data of UDCA-treated and untreated patients with FALD

	Total (*n* = 220)	Pre-matching			Propensity score matching		
		UDCA (–) (*n* = 107)	UDCA (+) (*n* = 113)	*p*-value [UDCA (–) vs. (+)]	UDCA (–) (*n* = 68)	UDCA (+) (*n* = 68)	*p*-value [UDCA (–) vs. (+)]
Age, years	26.1 (9.3–57.2)	25.9 (9.3–47.1)	26.7 (12.3–57.2)	0.07	26.5 (13.7–47.1)	27.7 (12.3–53.0)	0.35
Albumin, g/dL	4.6 (2.4–5.7)	4.6 (2.4–5.5)	4.6 (2.4–5.7)	0.43	4.6 (2.4–5.5)	4.6 (3.0–5.7)	0.30
Total bilirubin, mg/dL	1.1 (0.2–15.7)	1.0 (0.3–15.7)	1.2 (0.2–9.8)	0.34	1.2 (0.3–15.7)	1.2 (0.4–7.8)	0.88
Aspartate aminotransferase, U/L	25 (10–141)	23 (10–98)	26 (12–141)	0.16	24 (15–98)	25 (12–141)	0.61
Alanine transaminase, U/L	22 (7–318)	20 (7–148)	24 (9–318)	0.09	22 (7–148)	24 (9–318)	0.60
Gamma-glutamyl transferase, U/L	87 (17–435)	60 (17–311)	114 (19–435)	<0.01	87 (17–311)	96 (19–325)	0.28
PT%*	72.8 (29.4–100.0)	73.5 (29.4–96.9)	70.8 (37.2–100.0)	0.65	72.4 (38.8–90.6)	68.1 (37.2–100.0)	0.95
PT-INR*	1.14 (0.93–2.41)	1.14 (0.97–2.41)	1.14 (0.93–1.65)	0.79	1.16 (1.04–1.61)	1.18 (0.93–1.65)	0.79
Platelet count, ×10^4^/μL	15.8 (3.3–50.1)	16.1 (5.8–50.1)	15.7 (3.3–38.9)	0.66	15.7 (5.9–50.1)	15.3 (4.0–38.9)	0.38
BNP, pg/mL	57.8 (5.8–1,138.4)	48.8 (5.8–1,138.4)	69.6 (5.8–383.4)	0.60	58.6 (5.8–1,138.4)	69.0 (5.8–383.4)	0.59
Alpha-fetoprotein, ng/mL	4 (1–81,663)	4 (1–81,663)	4 (1–10,896)	0.60	4 (1–81,663)	4 (1–10,896)	0.38
Hyaluronic acid, ng/mL	47.0 (10.0–244.0)	47.0 (10.0–244.0)	47.0 (11.0–179.0)	0.71	67.0 (12.0–244.0)	51.0 (11.0–179.0)	0.23
Type IV collagen 7S, ng/mL	7.6 (0.9–14.7)	7.5 (3.5–12.0)	8.0 (0.9–14.7)	0.07	7.6 (3.5–12.0)	8.0 (4.9–12.0)	0.09
MELD-XI	10.59 (9.44–41.82)	9.59 (9.44–41.82)	11.58 (9.44–36.28)	0.17	11.78 (9.44–41.82)	11.58 (9.44–33.60)	0.72

Data are presented as median (range)

*BNP* brain natriuretic peptide, *FALD* Fontan-associated liver disease, *MELD-XI* model for end-stage liver disease excluding the international normalized ratio (INR), *PT* prothrombin time, *UDCA* ursodeoxycholic acid

^*^Patients treated with warfarin potassium (*n* = 146) were excluded

### Efficacy of UDCA treatment evaluated according to laboratory data

**Fig. 2:**
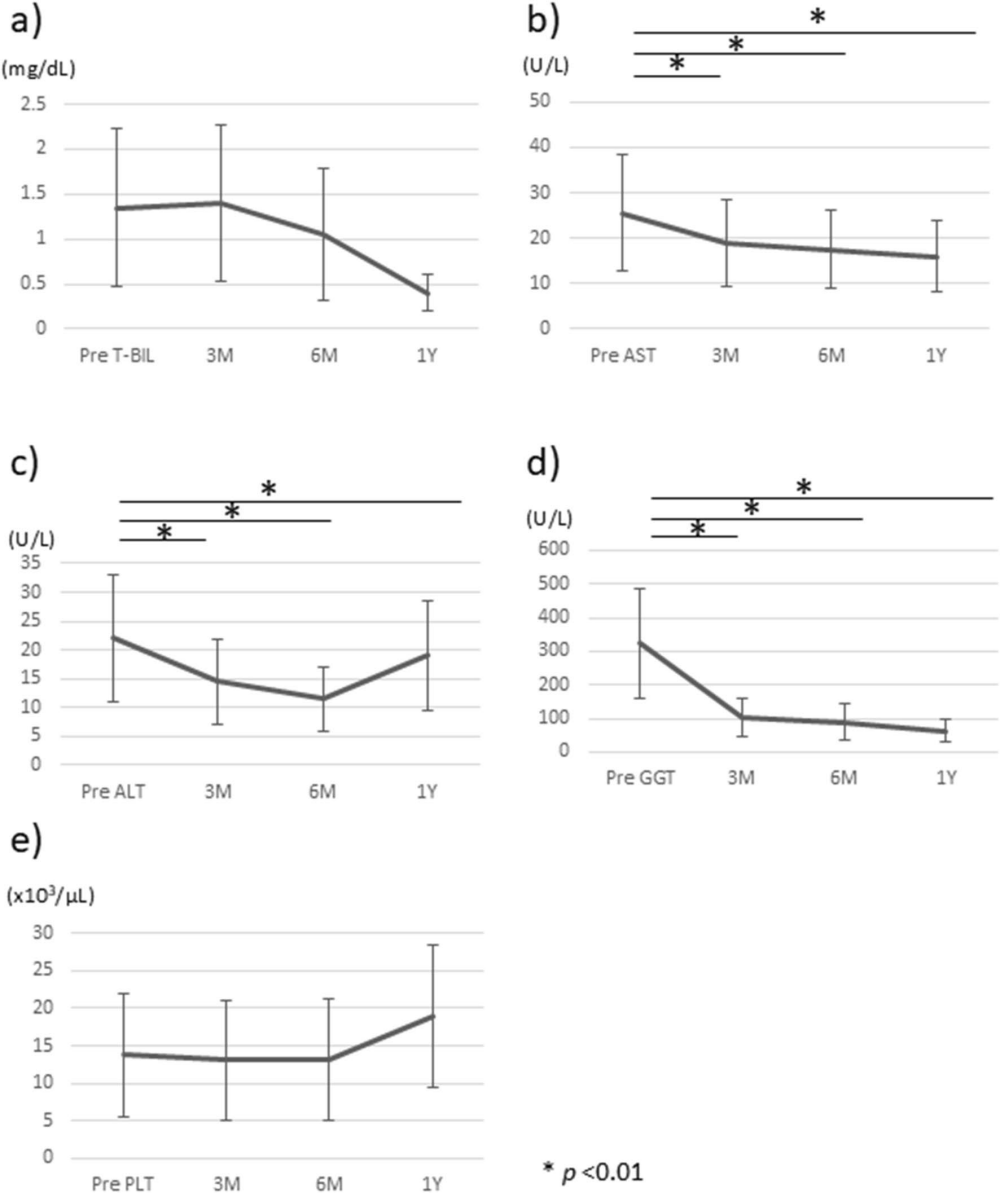
Changes in liver and biliary enzyme levels and platelet count following UDCA treatment. **a** Mean T-BIL levels were slightly decreased at 6 and 12 months after UDCA treatment; however, the change was not statistically significant. There were significant decreases in (**b**) AST levels, **c** ALT levels, and **d** GGT levels following UDCA treatment. **e** Platelet counts slightly increased 12 months after treatment but the change was not statistically significant. Data are presented as means with standard deviations. *ALT* alanine transaminase, *AST* aspartate aminotransferase, *GGT* gamma-glutamyl transferase, *M* months, *T-BIL* total bilirubin, *PLT* platelet count, *UDCA* ursodeoxycholic acid, *Y* year

We monitored changes in liver and biliary enzyme levels 3, 6, and 12 months after UDCA treatment. The mean serum levels of T-BIL were slightly lower at 6 and 12 months but the change was not statistically significant compared to the pretreatment levels (Fig. 2). By contrast, the AST, ALT, and GGT levels were significantly lower at all time points after treatment. The liver enzyme levels remained near normal for several years thereafter. The platelet counts were slightly higher after 1 year of treatment but the change was not statistically significant.

### Comparison of UDCA treatment in propensity-matched conditions

Given the differences in patient backgrounds, a propensity-matched analysis was performed to ensure comparability between the UDCA-treated and untreated groups. Matching was based on age at Fontan surgery, sex, GGT level, MELD-XI score, and warfarin use (Tables 1 and 3). After matching, the age and laboratory data were similar between UDCA-treated and untreated patients.

**Fig. 3:**
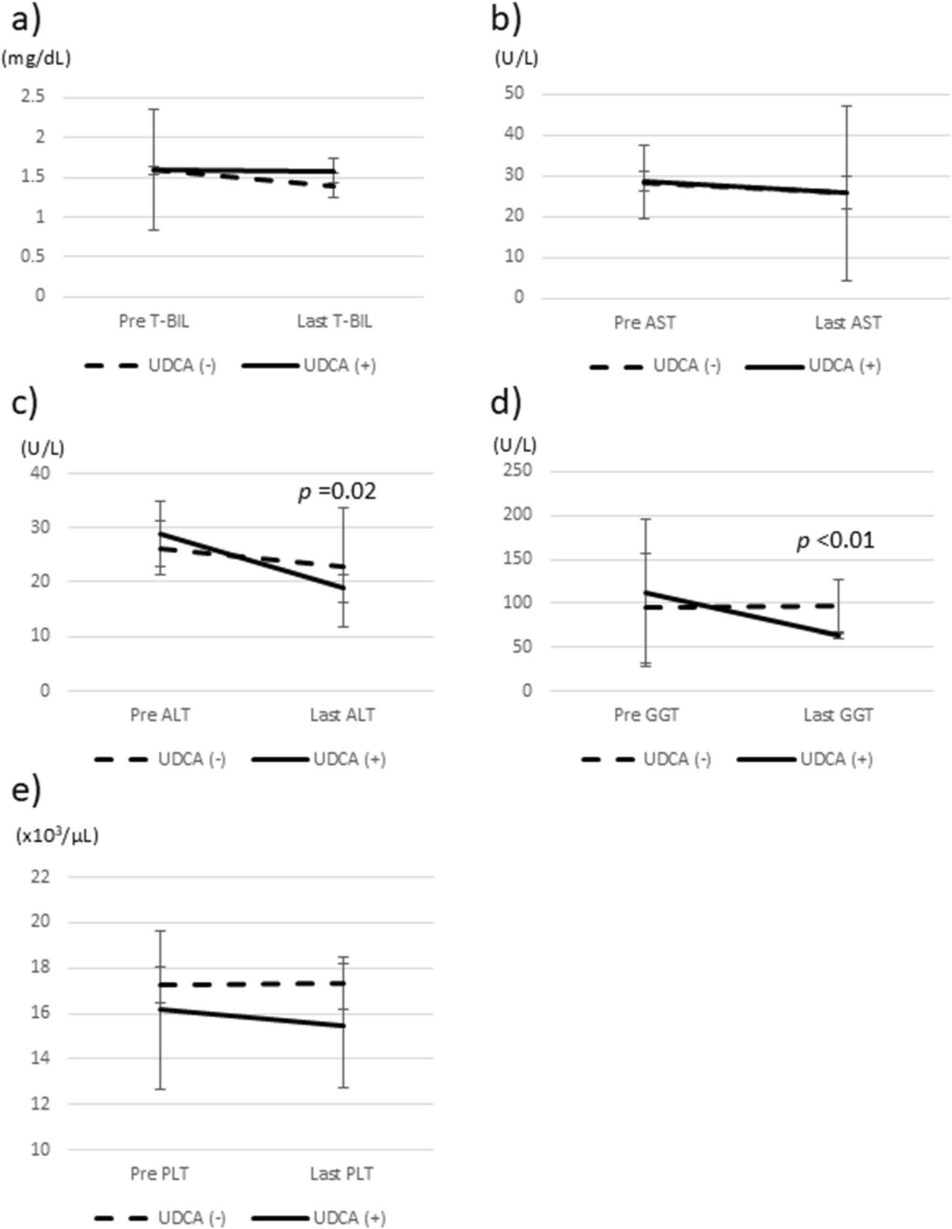
Comparison of liver and biliary enzyme levels between UDCA-treated and untreated patients with ≥6 months of follow-up. There were no significant differences in (**a**) T-BIL levels or (**b**) AST levels between UDCA-treated and untreated patients. There were significant reductions in (**c**) ALT levels and **d** GGT levels in treated patients vs. untreated patients. **e** Platelet counts did not significantly differ between the groups. Data are presented as means with standard deviations. *ALT* alanine transaminase, *AST* aspartate aminotransferase, *GGT* gamma-glutamyl transferase, *T-BIL* total bilirubin, *PLT* platelet count, *UDCA* ursodeoxycholic acid

Next, we compared the changes in liver and biliary enzyme levels between the matched groups (UDCA-treated group, *n* = 63; untreated group, *n* = 55). The treated group received treatment for a minimum follow-up period of 6 months, with an overall mean follow-up duration of 5.8 years (range, 0.5–20.9 years). The T-BIL and AST levels did not significantly differ between the two groups but ALT and GGT did, being significantly lower in treated patients. Platelet counts were not significantly different between the two groups (Fig. 3).

### Survival and HCC rates of patients with FALD stratified by UDCA treatment

**Fig. 4.**
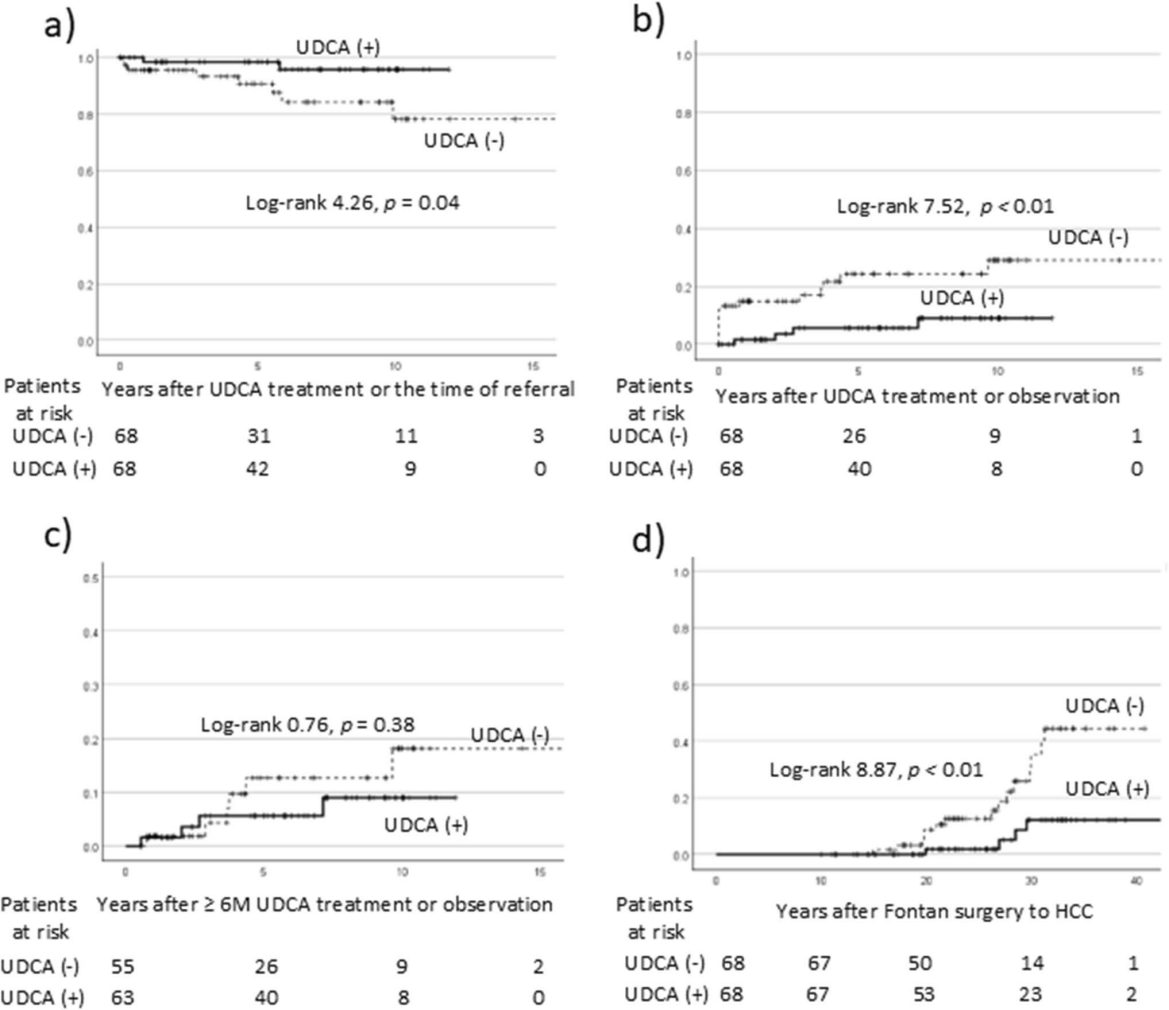
Survival and HCC incidence rates in patients with FALD stratified by UDCA treatment. **a** Kaplan–Meier survival curve for the total cohort. The 5-year survival rates were significantly higher in UDCA-treated patients than in untreated patients. **b** Kaplan–Meier curve showing the incidence of HCC in the total cohort. The incidence was significantly lower in treated patients. **c** Kaplan–Meier curve for the incidence of HCC in patients with ≥6 months of follow-up. The difference in HCC incidence between the groups was not statistically significant. **d** Kaplan–Meier curve for the time to HCC occurrence following Fontan surgery. The occurrence of HCC was significantly delayed in treated patients relative to untreated ones. *FALD* Fontan-associated liver disease, *HCC* hepatocellular carcinoma, *UDCA* ursodeoxycholic acid

In total, 10 patients (4.5%) died during the study period, with 2 deaths in the treated group and 8 in the untreated group. The causes of death were liver failure/HCC in 6 patients, sepsis in 2 patients, and heart failure/subarachnoid hemorrhage in 1 patient each. After propensity score matching, survival rates were analyzed using the Kaplan–Meier method (Fig. 4). The 5-year survival rate was 98.4% for treated patients and 90.7% for untreated patients, with a significant difference between the groups (*p* = 0.04).

HCC was observed in 4 UDCA-treated patients and in 15 untreated patients (Table 1). The 5-year incidence of HCC was significantly higher in the untreated group (24.2% vs. 5.6%, respectively (Fig. 4). Among patients followed ≥ 6 months (Fig. 1), the incidence of HCC increased in the untreated group; however, there were no significant differences between the groups (Fig. 4). When considering the timing of HCC occurrence post-Fontan surgery, the time to HCC occurrence was significantly longer in treated patients. This suggests that UDCA may reduce or delay HCC development. We analyzed the incidence of HCC in anticoagulant-treated versus untreated patients. HCC was observed in 12 anticoagulant-treated patients (12.2%) and in 7 untreated patients (18.4%), with no significant difference (*p* = 0.35).

Furthermore, Cox regression analysis was conducted to estimate the HR and 95% CI for the impact of UDCA treatment on HCC development, adjusting for potential confounders such as age at Fontan surgery, sex, GGT level, MELD-XI score, and warfarin use. UDCA treatment was negatively associated with HCC development, with an HR of 0.24 (95% CI, 0.081–0.737, *p* = 0.01).

In our Cox analysis on overall survival, HCC was strongly associated with poor outcomes (HR 11.05; 95% CI, 2.483–49.154; *p* < 0.01). Other significant factors included the MELD-XI score (HR 1.166; 95% CI, 1.064–1.277; *p* < 0.01) and age at Fontan surgery (HR 1.111; 95% CI, 1.023–1.206; *p* = 0.01). Although UDCA was potentially associated with improved overall survival (HR 0.202; 95% CI, 0.035–1.150; *p* = 0.07), GGT was not selected as a significant factor.

## Discussion

UDCA treatment significantly reduced the AST, ALT, and GGT levels starting from 3 months of treatment in patients with FALD. In addition, the treatment was associated with a significantly higher survival rate and a potential reduction in HCC development, suggesting that UDCA may be a beneficial therapeutic option for patients with FALD.

In patients with FALD, the GGT level is frequently elevated and serves as a diagnostic marker of liver damage. In our cohort, the median GGT level was 87 U/L (range, 17–435 U/L). Cellular GGT plays a key role in metabolizing extracellular reduced glutathione (GSH), and the serum level of GGT is considered a marker of oxidative stress, which can deplete GSH [23]. In chronic heart disease, an elevated GGT level is associated with major adverse cardiac events and cardiovascular mortality. In a previous study, we showed that the platelet count decreased more rapidly in patients with higher than lower GGT levels, and UDCA treatment was effective for reducing GGT levels [10]. This suggests that UDCA is a promising treatment option for FALD.

Although UDCA is commonly used for chronic liver diseases [16–18], its efficacy in patients with FALD has not been well established. In this study, UDCA was administered to 113 patients with FALD, with short-term treatment (up to 1 year) leading to significant reductions in GGT, AST, and ALT levels without severe side effects. During the follow-up period of 5.8 years (range, 0.5–20.9 years), UDCA continued to reduce the serum levels of liver enzymes. With the exception of the GGT levels, there were no significant differences in liver enzyme levels or platelet counts between patients who did and did not receive UDCA treatment.

A nationwide study in Japan reported a mortality rate of 0.19% (5 of 2,700 patients) [11]. In our cohort, the mortality rate was 4.5% (10 of 220 cases), indicating a higher mortality rate among patients with complicating FALD. The survival rate was significantly higher in UDCA-treated patients, and we speculated that HCC may be associated with mortality following the Fontan procedure [24].

Several studies have revealed that UDCA has an inhibitory effect on carcinogenesis, including HCC [25–28]. In previous papers, UDCA was reported to suppress HCC cell growth through inhibiting DLC1 (deleted in liver cancer 1), a tumor suppressor gene, and promoting protein degradation [25]. It has also been shown to induce apoptosis [26]. Another possible mechanism of UDCA about HCC suppression, it inhibits hypoxic HCC cell-induced angiogenesis [27]. However, whether UDCA directly prevents HCC development remains an open question that requires further investigation. In our study, HCC was significantly more common in untreated patients. However, there were no significant changes in the incidence of HCC regardless of UDCA treatment among patients with ≥6 months of UDCA treatment (*p* = 0.38). Over time, the incidence of HCC increased in untreated patients, suggesting that prolonged UDCA administration may effectively suppress carcinogenesis. When we analyzed the time to HCC occurrence following Fontan surgery, the occurrence of HCC was significantly delayed in UDCA-treated patients, indicating that UDCA may reduce or delay HCC development. However, the direct effect of UDCA on HCC suppression could not be determined.

Another potential factor in HCC suppression is the use of anticoagulants. Previous studies have shown that anticoagulant use reduces HCC development in patients with FALD [15]. In our cohort, UDCA-treated patients were more likely to be undergoing warfarin treatment. After matching for age, laboratory data, and warfarin use, HCC was still more common in untreated patients. To examine the possibility that HCC was inhibited by warfarin rather than UDCA, we analyzed the incidence of HCC in anticoagulant-treated versus untreated patients. HCC was observed in 12 anticoagulant-treated patients (12.2%) and in 7 untreated patients (18.4%), with no significant difference (*p* = 0.35). Moreover, Cox regression analysis for HCC development, adjusting for potential confounders (including warfarin use), showed that UDCA treatment was negatively associated with HCC development. This suggests that UDCA may be associated with a reduction in the incidence of HCC. HCC is strongly associated with survival in the patients with FALD and it might be improved the outcome.

This study had several limitations, including its single-center, retrospective design. We did not analyze the pathological improvements resulting from UDCA treatment, and the effects on fibrosis and HCC suppression require further verification through double-blind studies involving a larger number of patients and a longer follow-up period.

## Conclusions

UDCA treatment significantly reduced liver enzyme levels, including GGT, and slowed the progression of HCC in patients with FALD. UDCA may be a beneficial treatment option for this patient population.

## Abbreviations

ALT: Alanine transaminase
AST: Aspartate aminotransferase
BNP: Brain natriuretic peptide
FALD: Fontan-associated liver disease
GGT: γ-Glutamyl transpeptidase
HCC: Hepatocellular carcinoma
MELD-XI: Model for end-stage liver disease excluding the International normalized ratio
PT: Prothrombin time
T-BIL: Total bilirubin
UDCA: Ursodeoxycholic acid

## References

[1] Asrani SK, Asrani NS, Freese DK, et al. Congenital heart disease and the liver. Hepatology. 2012;56:1160–9. 10.1002/hep.25692. 22383293 10.1002/hep.25692

[2] Hilscher MB, Wells ML, Venkatesh SK, et al. Fontan-associated liver disease. Hepatology. 2022;75:1300–21. 10.1002/hep.32406. 35179797 10.1002/hep.32406

[3] Wu FM, Earing MG, Aboulhosn JA, et al. Predictive value of biomarkers of hepatic fibrosis in adult Fontan patients. J Heart Lung Transplant. 2017;36:211–9. 10.1016/j.healun.2016.07.011. 27592026 10.1016/j.healun.2016.07.011

[4] Elder RW, Parekh S, Book WM. More on hepatocellular carcinoma after the Fontan procedure. N Engl J Med. 2013;369:490. 10.1056/NEJMc1306854. 23902510 10.1056/NEJMc1306854

[5] Téllez L, Payancé A, Tjwa E, et al. EASL-ERN position paper on liver involvement in patients with Fontan-type circulation. J Hepatol. 2023;79:1270–301. 10.1016/j.jhep.2023.07.013. 37863545 10.1016/j.jhep.2023.07.013

[6] Ohfuji S, Tanaka A, Kogiso T, et al. Epidemiology of Fontan-associated liver disease in Japan: results from a nationwide survey in 2021. Hepatol Res. 2024. 10.1111/hepr.14040. 38526972 10.1111/hepr.14040

[7] Koenig G, Seneff S. Gamma-glutamyltransferase: a predictive biomarker of cellular antioxidant inadequacy and disease risk. Dis Markers. 2015;2015: 818570. 10.1155/2015/818570. 26543300 10.1155/2015/818570 PMC4620378

[8] Bulusu S, Sharma M. What does serum γ-glutamyltransferase tell us as a cardiometabolic risk marker? Ann Clin Biochem. 2016;53:312–32. 10.1177/0004563215597010. 26139450 10.1177/0004563215597010

[9] Shimizu M, Miyamoto K, Nishihara Y, et al. Risk factors and serological markers of liver cirrhosis after Fontan procedure. Heart Vessels. 2016;31:1514–21. 10.1007/s00380-015-0743-4. 26386570 10.1007/s00380-015-0743-4

[10] Kogiso T, Ogasawara Y, Taniai M, et al. Importance of gamma-glutamyl transferase elevation in patients with Fontan-associated liver disease. Hepatol Res. 2024. 10.1111/hepr.14093. 38985389 10.1111/hepr.14093

[11] Kuwabara M, Niwa K, Toyoda T, et al. Liver cirrhosis and/or hepatocellular carcinoma occurring late after the fontan procedure - a nationwide survey in Japan. Circ J. 2018;82:1155–60. 10.1253/circj.CJ-17-1053. 29445059 10.1253/circj.CJ-17-1053

[12] Sagawa T, Kogiso T, Sugiyama H, et al. Characteristics of hepatocellular carcinoma arising from Fontan-associated liver disease. Hepatol Res. 2020. 10.1111/hepr.13500. 32219953 10.1111/hepr.13500

[13] Inuzuka R, Nii M, Inai K, et al. Predictors of liver cirrhosis and hepatocellular carcinoma among perioperative survivors of the Fontan operation. Heart. 2023;109:276–82. 10.1136/heartjnl-2022-320940. 35768191 10.1136/heartjnl-2022-320940

[14] Kogiso T, Sagawa T, Taniai M, et al. Risk factors for Fontan-associated hepatocellular carcinoma. PLoS ONE. 2022;17: e0270230. 10.1371/journal.pone.0270230. 35714161 10.1371/journal.pone.0270230 PMC9205474

[15] Sakamori R, Yamada R, Tahata Y, et al. The absence of warfarin treatment and situs inversus are associated with the occurrence of hepatocellular carcinoma after Fontan surgery. J Gastroenterol. 2022;57:111–9. 10.1007/s00535-021-01842-8. 35064829 10.1007/s00535-021-01842-8

[16] Mitsuyoshi H, Nakashima T, Sumida Y, et al. Ursodeoxycholic acid protects hepatocytes against oxidative injury via induction of antioxidants. Biochem Biophys Res Commun. 1999;263:537–42. 10.1006/bbrc.1999.1403. 10491327 10.1006/bbrc.1999.1403

[17] Oka H, Toda G, Ikeda Y, et al. A multi-center double-blind controlled trial of ursodeoxycholic acid for primary biliary cirrhosis. Gastroenterol Jpn. 1990;25:774–80. 10.1007/BF02779195. 1980654 10.1007/BF02779195

[18] Makino I, Tanaka H. From a choleretic to an immunomodulator: historical review of ursodeoxycholic acid as a medicament. J Gastroenterol Hepatol. 1998;13:659–64. 10.1111/j.1440-1746.1998.tb00707.x. 9715413 10.1111/j.1440-1746.1998.tb00707.x

[19] Kogiso T, Tokushige K. Fontan-associated liver disease and hepatocellular carcinoma in adults. Sci Rep. 2020;10:21742. 10.1038/s41598-020-78840-y. 33303924 10.1038/s41598-020-78840-y PMC7728791

[20] Minagawa M, Ikai I, Matsuyama Y, et al. Staging of hepatocellular carcinoma: assessment of the Japanese TNM and AJCC/UICC TNM systems in a cohort of 13,772 patients in Japan. Ann Surg. 2007;245:909–22. 10.1097/01.sla.0000254368.65878.da. 17522517 10.1097/01.sla.0000254368.65878.da PMC1876960

[21] Shiina Y, Inai K, Sakai R, et al. Hepatocellular carcinoma and focal nodular hyperplasia in patients with Fontan-associated liver disease: characterisation using dynamic gadolinium ethoxybenzyl diethylenetriamine pentaacetic acid-enhanced MRI. Clin Radiol. 2023;78:e197–203. 10.1016/j.crad.2022.10.012. 36481111 10.1016/j.crad.2022.10.012

[22] Bertero L, Massa F, Metovic J, et al. Eighth Edition of the UICC Classification of Malignant Tumours: an overview of the changes in the pathological TNM classification criteria-What has changed and why? Virchows Arch. 2018;472:519–31. 10.1007/s00428-017-2276-y. 29209757 10.1007/s00428-017-2276-y

[23] Lee DH, Blomhoff R, Jacobs DR. Is serum gamma glutamyltransferase a marker of oxidative stress? Free Radic Res. 2004;38:535–9. 10.1080/10715760410001694026. 15346644 10.1080/10715760410001694026

[24] Alsaied T, Bokma JP, Engel ME, et al. Factors associated with long-term mortality after Fontan procedures: a systematic review. Heart. 2017;103:104–10. 10.1136/heartjnl-2016-310108. 28057809 10.1136/heartjnl-2016-310108

[25] Chung GE, Yoon JH, Lee JH, et al. Ursodeoxycholic acid-induced inhibition of DLC1 protein degradation leads to suppression of hepatocellular carcinoma cell growth. Oncol Rep. 2011;25:1739–46. 10.3892/or.2011.1239. 21455586 10.3892/or.2011.1239

[26] Xu Y, Luo Q, Lin T, et al. U12, a UDCA derivative, acts as an anti-hepatoma drug lead and inhibits the mTOR/S6K1 and cyclin/CDK complex pathways. PLoS ONE. 2014;9: e113479. 10.1371/journal.pone.0113479. 25486097 10.1371/journal.pone.0113479 PMC4259312

[27] Lin W, Li S, Meng Y, et al. UDCA Inhibits hypoxic hepatocellular carcinoma cell-induced angiogenesis through suppressing HIF-1α/VEGF/IL-8 intercellular signaling. Front Pharmacol. 2021;12: 755394. 10.3389/fphar.2021.755394. 34975472 10.3389/fphar.2021.755394 PMC8714963

[28] Režen T, Rozman D, Kovács T, et al. The role of bile acids in carcinogenesis. Cell Mol Life Sci. 2022;79:243. 10.1007/s00018-022-04278-2. 35429253 10.1007/s00018-022-04278-2 PMC9013344

